# SiaA/D Interconnects c-di-GMP and RsmA Signaling to Coordinate Cellular Aggregation of *Pseudomonas aeruginosa* in Response to Environmental Conditions

**DOI:** 10.3389/fmicb.2016.00179

**Published:** 2016-02-26

**Authors:** Brendan Colley, Verena Dederer, Michael Carnell, Staffan Kjelleberg, Scott A. Rice, Janosch Klebensberger

**Affiliations:** ^1^Centre for Marine Bio-Innovation, School of Biotechnology and Biomolecular Sciences, University of New South WalesSydney, NSW, Australia; ^2^Institute of Technical Biochemistry, University of StuttgartStuttgart, Germany; ^3^Biomedical Image Facility, Mark Wainwright Analytical Centre, University of New South WalesSydney, NSW, Australia; ^4^Singapore Centre for Environmental Life Sciences Engineering and School of Biological Sciences, Nanyang Technological University, SingaporeSingapore

**Keywords:** c-di-GMP signaling, RsmA, Pseudomonas aeruginosa, cellular aggregation, biofilm, SiaA, SiaD, CupA fimbriae

## Abstract

*Pseudomonas aeruginosa* has emerged as an important opportunistic human pathogen that is often highly resistant to eradication strategies, mediated in part by the formation of multicellular aggregates. Cellular aggregates may occur attached to a surface (biofilm), at the air-liquid interface (pellicle), or as suspended aggregates. Compared to surface attached communities, knowledge about the regulatory processes involved in the formation of suspended cell aggregates is still limited. We have recently described the SiaA/D signal transduction module that regulates macroscopic cell aggregation during growth with, or in the presence of the surfactant SDS. Targets for SiaA/D mediated regulation include the Psl polysaccharide, the CdrAB two-partner secretion system and the CupA fimbriae. While the global regulators c-di-GMP and RsmA are known to inversely coordinate cell aggregation and regulate the expression of several adhesins, their potential impact on the expression of the *cupA* operon remains unknown. Here, we investigated the function of SiaA (a putative ser/thr phosphatase) and SiaD (a di-guanylate cyclase) in *cupA1* expression using transcriptional reporter fusions and qRT-PCR. These studies revealed a novel interaction between the RsmA posttranscriptional regulatory system and SiaA/D mediated macroscopic aggregation. The RsmA/*rsmY*/*Z* system was found to affect macroscopic aggregate formation in the presence of surfactant by impacting the stability of the *cupA1* mRNA transcript and we reveal that RsmA directly binds to the *cupA1* leader sequence *in vitro*. We further identified that transcription of the RsmA antagonist *rsmZ* is controlled in a SiaA/D dependent manner during growth with SDS. Finally, we found that the *siaD* transcript is also under regulatory control of RsmA and that overproduction of RsmA or the deletion of *siaD* results in decreased cellular cyclic di-guanosine monophosphate (c-di-GMP) levels quantified by a transcriptional reporter, demonstrating that SiaA/D connects c-di-GMP and RsmA/*rsmY*/*Z* signaling to reciprocally regulate cell aggregation in response to environmental conditions.

## Introduction

It is now well established that most bacteria can form multicellular aggregates and that this is believed to represent the dominant mode of microbial life (Costerton et al., 1995; Hall-Stoodley et al., 2004). Cellular aggregates can occur attached to a surface as biofilms, as a pellicle at the air-liquid interface, or as suspended aggregates of cells. The evolutionary and ecological success of the multicellular lifestyle has often been attributed to the increased resistance of the resident cells compared to their planktonic free living counterparts (Davey and O’Toole, 2000; Hall-Stoodley et al., 2004). The increased resistance of cellular aggregates can impact human health, for example by allowing the persistence of bacteria in health-care settings despite the use of hygiene regimens, which provides a potential reservoir for the transmission of infections (Kerr and Snelling, 2009; Asghari et al., 2013; Williams et al., 2013). *Pseudomonas aeruginosa* has emerged as an opportunistic pathogen for patients with compromised immunity, such as those with the genetic disorder cystic fibrosis (Bjarnsholt, 2013). In the context of chronic lung infections of cystic fibrosis patients, *P. aeruginosa* is thought to survive predominantly as cell aggregates growing in mucus that are not associated with any surface (Bjarnsholt et al., 2009). In addition, recent studies indicate that aggregation is commonly observed for *P. aeruginosa* growing in liquid environments (Schleheck et al., 2009).

The reciprocal regulation of cellular aggregation mediated by the RsmA and c-di-GMP global regulatory systems has been well documented, with high levels of c-di-GMP leading to a sessile lifestyle, in contrast to RsmA, which promotes planktonic physiology (Hengge, 2009; Timmermans and Van Melderen, 2010; Romling et al., 2013; Vakulskas et al., 2015). Both regulators can function at multiple levels of gene expression either directly or by influencing additional regulators. C-di-GMP has been found to specifically bind to mRNA riboswitches and a range of proteins to exert its regulatory potential (Amikam and Galperin, 2006; Hickman and Harwood, 2008; Sudarsan et al., 2008; Boehm et al., 2010; Chambers et al., 2014; Trampari et al., 2015). The RsmA protein acts directly by binding to the mRNA of target genes, typically by binding to a consensus sequence (Dubey et al., 2005) overlapping the Shine-Dalgarno sequence (SD), which prevents ribosome assembly, reducing translation, and most often resulting in more rapid degradation of the transcript by RNAses (Timmermans and Van Melderen, 2010; Vakulskas et al., 2015). The GacA/S two-component system is the best characterized regulatory system that influences the activity of RsmA, where GacA/S controlled *rsmY*/*Z* sRNA expression leads to their binding and functional sequestration of the RsmA protein (Goodman et al., 2004; Kay et al., 2006; Sorger-Domenigg et al., 2007; Brencic et al., 2009).

The RsmA/*rsmY*/*Z* and c-di-GMP pathways can affect the expression of a large number of target genes in *P. aeruginosa*, and are predicted to display significant cross-talk (Coggan and Wolfgang, 2012). Interestingly, recent evidence indicates that *rsmY* or *rsmZ* regulation is not limited to the GacA/S two-component system, suggesting that additional mechanisms influence the regulation of genes targeted by RsmA in response to certain environmental conditions. This system has been shown to be affected by oxygen concentration (*via* the Anr responsive NarL transcription factor; O’Callaghan et al., 2011), changes in biofilm growth stage (*via* BfiR; Petrova and Sauer, 2010), envelope stress resulting from loss of the OprF porin (possibly *via* SigX; Bouffartigues et al., 2015) as well as controlling virulence factor production (through RetS and the hybrid sensor PA1611; Kong et al., 2013). Thus, the RsmA/*rsmY/Z* regulatory system impacts many aspects of growth and development of *P. aeruginosa*.

In the context of cellular aggregation, c-di-GMP and RsmA are well known to be involved in the expression of several cell surface adhesins including the Psl polysaccharide and the CdrAB two-partner secretion system (Burrowes et al., 2006; Brencic and Lory, 2009; Borlee et al., 2010; Irie et al., 2010). Accordingly, either mutation of *rsmA* or artificially increasing c-di-GMP levels leads to Psl and CdrAB dependent autoaggregation irrespective of the environmental conditions (Starkey et al., 2009; Borlee et al., 2010; Irie et al., 2010, 2012). Interestingly, the CupA fimbriae are also known to be involved in cellular aggregation at surfaces (Meissner et al., 2007), although the genes of the *cupA* operon have not previously been shown to be influenced by RsmA, and the evidence that c-di-GMP regulates the *cupA* operon is conflicting (Meissner et al., 2007; Starkey et al., 2009).

We have recently described that macroscopic cellular aggregation of *P. aeruginosa* can be induced by exposure to sub-inhibitory concentrations of the surfactant sodium dodecyl sulfate (SDS) and that these aggregated cells display an increased resistance to additional stresses (Klebensberger et al., 2006, 2007). By mutational analysis and phenotypic characterisation, SiaA/D was shown to play a central role in this process in a c-di-GMP dependent manner (Klebensberger et al., 2009). The functions of both the putative ser/thr dependent phosphatase SiaA and the di-guanylate cyclase (DGC) SiaD were shown to be required for macroscopic aggregate formation during growth with SDS by regulating the expression of a core set of genes including those encoding the CupA fimbriae (Klebensberger et al., 2009). In the present study, we show that RsmA signaling is a negative regulator of macroscopic aggregation during growth with SDS, and that *cupA1* represents a novel target for RsmA binding. Further, we provide strong evidence that this is achieved by SiaA/D dependent transcriptional regulation of *rsmZ* and that the *siaABCD* operon itself is under negative feedback regulation of RsmA. From these data we conclude that SiaA/D connects the c-di-GMP and RsmA networks to reciprocally coordinate the aggregation of cells in response to environmental conditions.

## Materials and Methods

### Bacterial Strains and Growth Conditions

*Pseudomonas aeruginosa* and *Escherichia coli* strains (Supplementary Table S1) were maintained on Lysogeny Broth (LB) (Bertani, 1951) solid medium (1.5% agar w/v) and routinely cultured in 10 mL LB medium in 50 mL Falcon tubes (Greiner Bio-one) with shaking at 200 rpm and incubation at 37°C (Ratek Orbital Mixer Incubator). Where appropriate, antibiotics were added at the following concentrations for maintenance or selection respectively: 50 or 100 μg mL^-1^ carbenicillin, 15 or 30 μg mL^-1^ gentamycin, 10 or 20 μg mL^-1^ tetracycline for *E. coli*, and 50 or 200 μg mL^-1^ carbenicillin, 30 or 120 μg mL^-1^ gentamycin, 20 or 200 μg mL^-1^ tetracycline for *P. aeruginosa*. For *cupA*::*lacZ* experiments, 10 μg mL^-1^, gentamicin and 10 μg mL^-1^ tetracycline was added to the growth medium for plasmid maintenance.

### Strain and Plasmid Construction

The E79tv2 phage was used to construct mutant strains by transferring mutant alleles into the wild-type PAO1 background used in this study as previously described (Malone et al., 2010). The PW8792 strain was obtained from the University of Washington Genome Centre and used as the source of the tetracycline resistant *cdrB* mutation. The resulting Δ*cdrB* strain was subsequently cured of the antibiotic resistance cassette by Cre/*loxP* recombination using the pCre1 plasmid (Jacobs et al., 2003). The MPAO1_KO*cupA3* and MPAO1_KO*pslBCD* strains (Kirisits et al., 2005; Starkey et al., 2009) were kindly provided by the Parsek lab (University of Washington, USA) and used as the sources of gentamycin resistant *cupA3* and *psl* mutations respectively. The resulting Δ*cupA3* and Δ*psl* strains were subsequently cured of the antibiotic resistance cassettes by FLP/*FRT* recombination using the pFLP2 plasmid (Hoang et al., 1998). The arabinose inducible *rsmA* allele was inserted at the neutral *att*Tn7 site of the *P. aeruginosa* chromosome by transposition using miniTn7T[P_BAD_::*rsmA*] (kindly provided by Tina Jäger from the Biozentrum Basel) and the pTNS2 plasmid (Choi et al., 2005). A detailed description for the construction of the pBBR[*siaD*], pUC18[*cupA*], pUC19[*lolB*], pUC19[*rsmZ*], pBAD33[RsmA-His_6_] plasmids and the primers used (Supplementary Table S2) is provided in the Supplementary Material.

### Growth and Cell Suspension Experiments

Growth experiments were performed in a modified M9 minimal medium (Klebensberger et al., 2006). Overnight cultures of *P. aeruginosa* strains grown in LB medium were inoculated into 2 mL M9 medium at an OD_600_ = 0.01 in 12 well microtitre plates (Corning) supplemented with either 10 mM succinate or 3.5 mM SDS and incubated at 30°C with shaking at 200 rpm for 18 h (Ratek orbital mixer incubator).

For cell suspension experiments, LB overnight cultures were used to inoculate 100 mL of M9 medium with an OD_600_ = 0.01 in a 500 mL Erlenmeyer flask. The medium was supplemented with either 10 mM succinate or 3.5 mM SDS as a growth substrate and incubated at 30°C with shaking at 200 rpm to the late-logarithmic phase of growth. Subsequently, the cells were washed twice with 10 mL M9 medium with centrifugation for 2 min at 10,000 × *g* at room temperature (Beckman Avanti J25-I), re-suspended in 1 mL M9 and used to generate cell suspensions with an OD_600_ = 1. Cell suspensions were supplemented with 10 mM succinate or 3.5 mM SDS, transferred to 12 well microtitre plates (Corning) in 2 mL samples and incubated as indicated below. Where appropriate, L-arabinose (2% final concentration) was included in both the 100 mL pre-culture and the cell suspension experiment. After incubation, images were acquired from growth and cell suspension experiments using an Umax Powerlook 1000 flatbed scanner with V4.71 software in a dark room. Images were normalized to the first image scanned using this experimental format by using Adobe Photoshop CS5 Software by using the ‘match color’ image adjustment. Experiments were performed multiple times (*n* ≥ 3) and a representative image is shown.

### Quantification of Macroscopic Aggregation

Scanned images of cultures grown in 12 well plates were analyzed using ImageJ (Schneider et al., 2012). Images were preprocessed by converting to 8-bit grayscale images and applying a rolling ball background subtraction to correct for the uneven background present within each well. A radius of 11 pixels was observed to retain aggregate structures yet flatten out the background suitable for thresholding. To remove bias, thresholds were determined automatically using an implementation of Huang’s thresholding (Huang and Wang, 1995) already available in ImageJ. Each well was then measured using ‘Analyze Particles’ with measurements restricted to circular regions of interest of consistent size, each positioned in the center to avoid any artifacts resulting from the edges of the wells. Individual data (circles) are shown together with the mean value of the three biological replicates (bars).

### *cupA1::lacZ* Transcriptional Reporter Studies

Cell suspensions for *P. aeruginosa* strains harboring the *cupA1*::*lacZ* transcriptional promoter fusion (Vallet et al., 2004) were prepared as described above. Following 1 h incubation at 30°C with shaking at 200 rpm, strains were harvested by centrifugation for 30 s at 16,060 × *g* (Heraues Biofuge Pico), washed twice in 1 mL fresh M9 medium, resuspended in 1 mL M9 medium and lysed with ∼65 mg of Cellytic express (Sigma) at 37°C in a water bath for 15 min with occasional mixing. Intact cells and cell debris were removed from the crude cell extracts by centrifugation for 1 min at 16,060 × *g* (Heraues Biofuge Pico) and 800 μL of the supernatant was transferred to a fresh Eppendorf tube and stored at -20°C for further analysis.

Beta-galactosidase (β-galactosidase) activity was measured based on the hydrolysis of ortho-nitrophenyl-β-galactoside (ONPG). For activity measurements, 20 μL of cell extract was incubated with 80 μL of β-galactosidase buffer (60 mM Na_2_HPO_4_, 40 mM NaH_2_PO_4_ and 30 mM β-mercaptoethanol, pH 7) in a 96 well plate (Sarstedt, flat bottom) at 30°C for 10 min. Immediately following this incubation, the reaction was started by the addition of 20 μL ONPG from a freshly prepared 4 μg mL^-1^ stock and incubated at 30°C. Reactions were stopped at specific time points by the addition of 50 μL of 1 M Na_2_CO_3_ solution and the absorbance measured at 420 nm in a plate reader (Perkin Elmer; Wallac Victor^2^). The concentration of ortho-nitrophenol (ONP), the enzymatic product formed by the hydrolysis of ONPG due to β-galactosidase activity, was calculated from a freshly prepared calibration curve. Specific β-galactosidase activity was determined by normalizing the values of β-galactosidase activity to the total protein content of the corresponding cell extract measured by the Bicinchoninic Acid (BCA) assay (Pierce), and is expressed as nmol ONPG min^-1^ mg^-1^ total protein. Individual data (circles) are shown together with the mean value of the three biological replicates (bars).

### Reporter Assays Using *cdrA, rsmY* and *rsmZ* Transcriptional *gfp* Fusions

To quantify intracellular c-di-GMP levels or the transcriptional activity of the sRNAs *rsmZ*/*Y*, *P. aeruginosa* strains harboring the CdrA::*gfp*^c^, *rsmZ*::*gfp* or *rsmY*::*gfp* fusion constructs (Rybtke et al., 2012; Chua et al., 2014) were grown in 10 mL LB at 30°C with shaking at 200 rpm for 10 h (Ratek orbital mixer incubator). After incubation, the cells were washed twice in 1 mL M9 and used to inoculate either 10 mL M9 medium containing either 10 mM succinate or 3.5 mM SDS with an OD_600_ = 0.01. Succinate cultures were grown for 9 h, while SDS cultures were grown for 18 h with incubation at 30°C and shaking at 200 rpm. These cultures were then washed twice in 10 mL M9 medium, resuspended in 1 mL M9 without carbon source and adjusted to an OD_600_ = 0.5. These cell suspensions were inoculated in 2 mL aliquots in 12 well plates and grown at 30°C shaking at 200 rpm for 30 min.

To quantify the CdrA::*gfp*^c^ reporter activity, fluorescence was measured in a plate reader using an excitation/emission of 485/535 nm with constant voltage for 1 s and the use of a large emission aperture (Perkin Elmer, Wallac victor^2^). The samples were normalized by the initial optical density and experiments consisted of six biological samples that were repeated as two technical replicates to ensure reproducibility of the data. Individual data (circles) are shown together with the mean value of the six biological replicates (bars).

To quantify the *rsmY* and *rsmZ* transcriptional reporter activity, each sample was passed through a 25 gauge syringe three times before the samples were lysed with Cellytic B reagent according to the manufacturer’s instructions (Sigma). The total protein content and the GFP signal of each sample was then measured with a Nanodrop (ND1000) using the protein determination (absorbance at 280 nm with baseline correction) and Alexaflour 488 (absorbance at 488 nm with dye slope correction) label quantification functions, respectively (Nanodrop v3.12 software). Finally, the relative GFP signal for each sample was determined by dividing the absorbance value at 488 nm by the corresponding total protein content. Experiments were performed in three biological replicates, each performed as two technical replicates. Individual data (circles) are shown together with the mean value of the three biological replicates (bars).

### mRNA Structural Prediction, mRNA Decay, and mRNA Abundance Experiments

mRNA structural prediction was performed using version 2.3 of the mFOLD web server (Zuker, 2003). Initially, sequences were scanned for the presence of the SELEX derived RsmA binding consensus ^A^/_U_CANGGANG^U^/_A_ (Dubey et al., 2005), and also for the presence of the primary and secondary RsmA binding sites used in the algorithm for predicting novel RsmA targets (Kulkarni et al., 2014). For the analysis of the *cupA1* leader sequence, the 200 nucleotides upstream and including the AUG start codon was used for analysis. For the analysis of the *siaA* leader sequence a 93 nt sequence from the previously described transcriptional start site from the AmrZ promoter (Jones et al., 2014) up to and including the AUG start codon and was used. mRNA secondary structures were predicted at 30°C using the mFOLD web server (version 2.3 energies) with standard settings, and the most stable structure according to the free energy (ΔG) predictions is presented.

The mRNA decay experiments were performed by generating cell suspensions of an OD_600_ = 0.5 as described above. These cell suspensions were incubated for 1 h at 30°C with shaking at 200 rpm (Ratek orbital mixer incubator), and then 300 μg mL^-1^ rifampicin was added to prevent *de novo* mRNA synthesis. Samples were taken immediately, and following 10, 20, and 30 min incubation at 30°C with shaking at 200 rpm. Samples were harvested by centrifugation for 30 s at 16,060 × *g* (Heraues Biofuge Pico) and washed once in 1 mL ice cold M9 before being treated with 1 mL RNAlater bacterial mRNA stabilization reagent according to the manufacturer’s instructions (Qiagen). Total RNA was extracted from the samples by using an RNeasy Mini Kit (Qiagen) according to the manufacturer’s instructions, including the use of the optional on-column DNAse treatment with RNAse free DNAse (Qiagen). Purified RNA was subjected to a second DNAse treatment with a Turbo DNAse kit (Ambion) to remove residual traces of genomic DNA. RNA samples were quantified with a NanoDrop (ND-1000) and random RNA samples were selected for Bioanalyser analysis to validate RNA integrity and accuracy of NanoDrop measurements (Agilent 2100 Electrophoresis Bioanalyser; Ramaciotti centre, UNSW) (available on request). cDNA was synthesized from 500 ng total RNA with an iScript cDNA Synthesis Kit using random hexamers according to the manufacturer’s instructions (Bio-rad). qPCR was performed in a black hard-shell 96-well plate with white wells (Bio-rad) in a CFX-1000 machine (Bio-rad) using Evagreen supermix (Bio-rad), 0.5 μL cDNA, 300 nM each primer in a final reaction volume of 25 μL. Each sample was measured in triplicate, and a cDNA synthesis reaction performed without the addition of reverse transcriptase enzyme was included as a control for the detection of gDNA. The reaction conditions were as follows; 3 min at 98°C for 1 cycle, followed by 60°C for 5 s, 72°C for 10 s and data acquisition, 98°C for 10 s for 40 cycles, and a melt curve performed from 60°C to 98°C with 0.5°C intervals. CFX1000 software was used to calculate cycle threshold (Ct) values, and data was analyzed according to the 2^-ΔCt^ method for mRNA decay analysis (Livak and Schmittgen, 2001). Individual data (circles) are shown together with the mean value of the three biological replicates (bars).

The same experimental format described for the mRNA decay experiments was used to quantify the relative abundance of mRNA, with the exception that following the 1 h incubation of cell suspensions total RNA was harvested immediately for analysis. Samples were processed as described above, and data was analyzed by the 2^-ΔΔCt^ method (Livak and Schmittgen, 2001) with the *proC* housekeeping gene used for normalization. Individual data (circles) are shown together with the mean value of the three biological replicates (bars).

### Surface Plasmon Resonance

RsmA binding studies were performed with purified RsmA-His6 protein and *in vitro* generated RNAs of *rsmZ*, *lolB*, *cupA1* and *cupA1[AAA]* using surface plasmon resonance measurements on a BIACore 3000 system equipped with a NTA sensor chip (GE Healthcare). Detailed information about the experimental procedure for protein purification, *in vitro* transcription, BIACore measurements and the data analysis can be found in the Supplementary Material.

### Statistical Methods

Bar graphs represent the mean value of all available biological replicates and are accompanied with the individual data points. Statistical significance was determined using a two-tailed, unpaired student *t*-test, with a *p*-value ≤0.05 considered significant.

## Results

### Essential Components of Aggregate Formation

**FIGURE 1:**
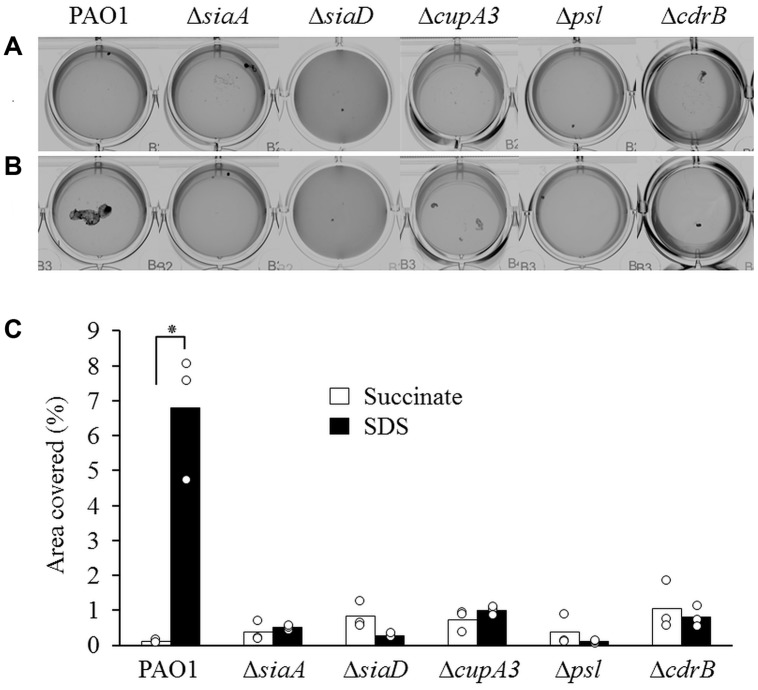
**Phenotypic characterisation of *Pseudomonas aeruginosa* and isogenic mutant strains during growth with 10 mM succinate (A) or 3.5 mM SDS (B). (C)** Quantification of the percentage of area covered by macroscopic aggregates of cells during growth with succinate (*white bars*) or SDS (*black bars*) from different strains. Bars represent the mean value from three individual experiments and are accompanied by the individual data points. Statistical significance is indicated (^∗^*p* = 0.003).

Previous studies have demonstrated that the putative ser/thr phosphatase SiaA and the DGC SiaD are essential for macroscopic aggregate formation during growth with, or in the presence of the surfactant SDS (Klebensberger et al., 2006, 2009). SiaA harbors a HAMP domain characteristic of sensory proteins that functions to link sensory perception with an intracellular response, such as the predicted phosphatase activity of its N-terminal PP2C ser/thr phosphatase domain. Further genetic analysis identified the CupA fimbriae, the CdrAB two-partner secretion system, and the Psl polysaccharide as regulatory targets for SiaA/D (Klebensberger et al., 2009). In this study, we used the Δ*siaA* and Δ*siaD* mutants to validate our experimental conditions and constructed defined mutations of *cupA3*, the *psl* operon, and *cdrB* to confirm their role in macroscopic aggregation during growth with SDS (**Figures 1A,B**). The wild-type strain PAO1 produced macroscopic aggregates during growth with SDS as previously described (Klebensberger et al., 2006). In contrast, mutation of *siaA*, *siaD*, *cupA3*, *psl*, or *cdrB* eliminated this phenotype, confirming their essential role in macroscopic aggregation. Quantification analysis revealed that growth with SDS increased the area of coverage by macroscopic aggregates 58 fold for the wild-type strain compared to growth with succinate (**Figure 1C**). In contrast, none of the, Δ*siaA*, Δ*siaD*, Δ*cupA3*, *psl*, or Δ*cdrB* mutant strains showed an increase in aggregation during growth with SDS.

### The GGEEF Active Site Motif of SiaD is Required for Aggregate Formation

**FIGURE 2:**
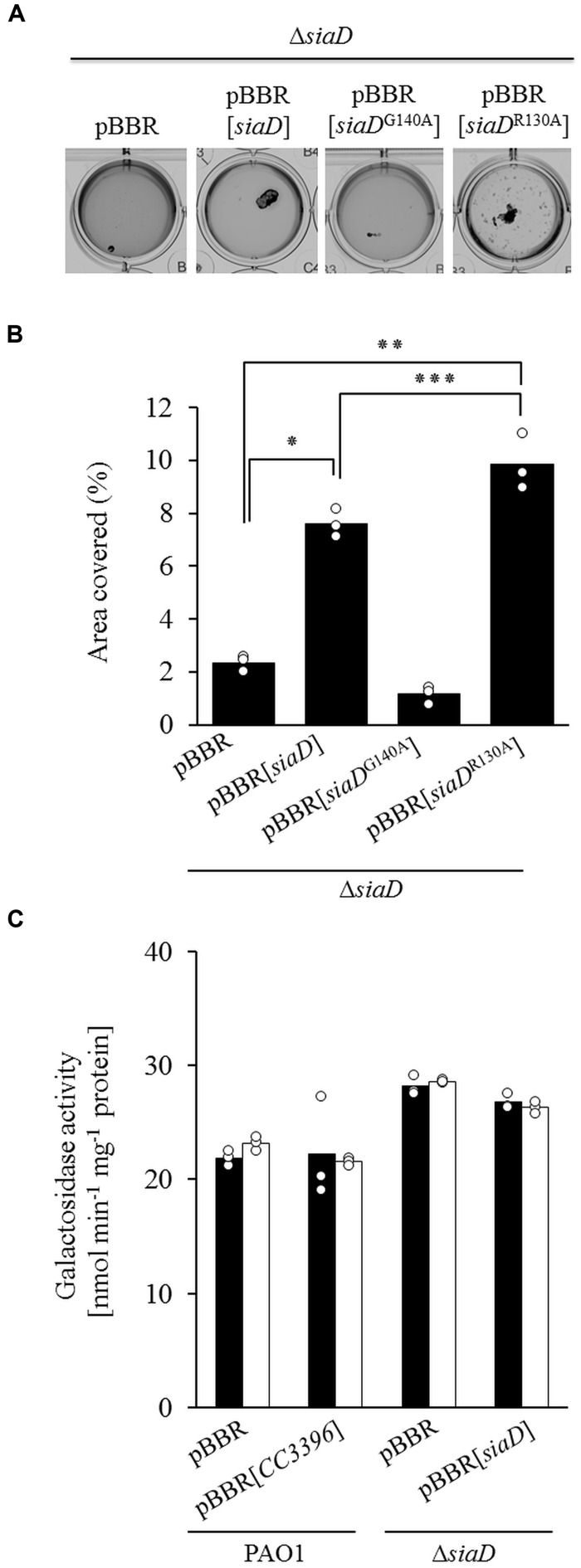
**(A)** Phenotypic analysis of the ability for the pBBR empty vector, the native *siaD* gene, and the mutated A-site *siaD*^G140A^ or I-site *siaD*^R130A^ alleles to restore aggregation to the Δ*siaD* mutant during growth with 3.5 mM SDS. **(B)** Quantitative analysis of the percentage of area covered by macroscopic aggregates of different strains. **(C)** Transcription of *cupA1* assessed with a *cupA::lacZ* reporter for the PAO1 pBBR plasmid control, the PAO1 pBBR[CC3396] strain encoding a functional c-di-GMP specific phosphodiesterase from *Caulobacter crescentus*, the Δ*siaD* mutant, and Δ*siaD* pBBR[*siaD*] complemented strain during growth in 10 mM succinate (*black bars*) or 3.5 mM SDS (*white bars*). Bars represent the mean value from three individual experiments and are accompanied by the individual data points. Statistical significance is indicated (^∗^*p* < 0.001, ^∗∗^*p* < 0.001, ^∗∗∗^*p* = 0.03).

Macroscopic aggregate formation involves c-di-GMP and the DGC SiaD (Klebensberger et al., 2009). This protein harbors both a GGEEF active-site motif (A-site) characteristic of c-di-GMP biosynthesis and a conserved RXXD motif (I-site) characteristic of c-di-GMP binding and allosteric regulation. Site specific mutagenesis was used to produce two *siaD* alleles with either a non-functional A-site (*siaD*^G140A^) or I-site (*siaD*^R130A^), and the resulting constructs were tested for their ability to complement the Δ*siaD* mutant (**Figure 2A**). Macroscopic aggregation assays and their subsequent quantitative analysis indicated that growth with SDS increased the area covered by macroscopic aggregates of the Δ*siaD* mutant expressing the native *siaD* gene or the mutant I-site allele *siaD*^R130A^, but not for the non-functional A-site allele *siaD*^G140A^ (**Figure 2B**). In addition, the observed increase was greater for cultures of the Δ*siaD* mutant expressing the defective I-site allele *siaD*^R130A^ compared to the strain complemented with the native *siaD* allele (9.85 vs. 7.60%).

### Regulation of *cupA* Fimbriae

Our previous transcriptomics based approach indicated that *cupA1* transcripts were 19 fold more abundant in wild-type cells during SDS exposure compared to cells grown with only succinate, and also when compared to the Δ*siaD* mutant grown with SDS (12 fold difference) (Klebensberger et al., 2009). To investigate a potential SiaA/D dependent transcriptional regulation of *cupA1*, we used a previously described functional *cupA1*::*lacZ* reporter (**Figure 2C**) (Vallet et al., 2004).

**FIGURE 3:**
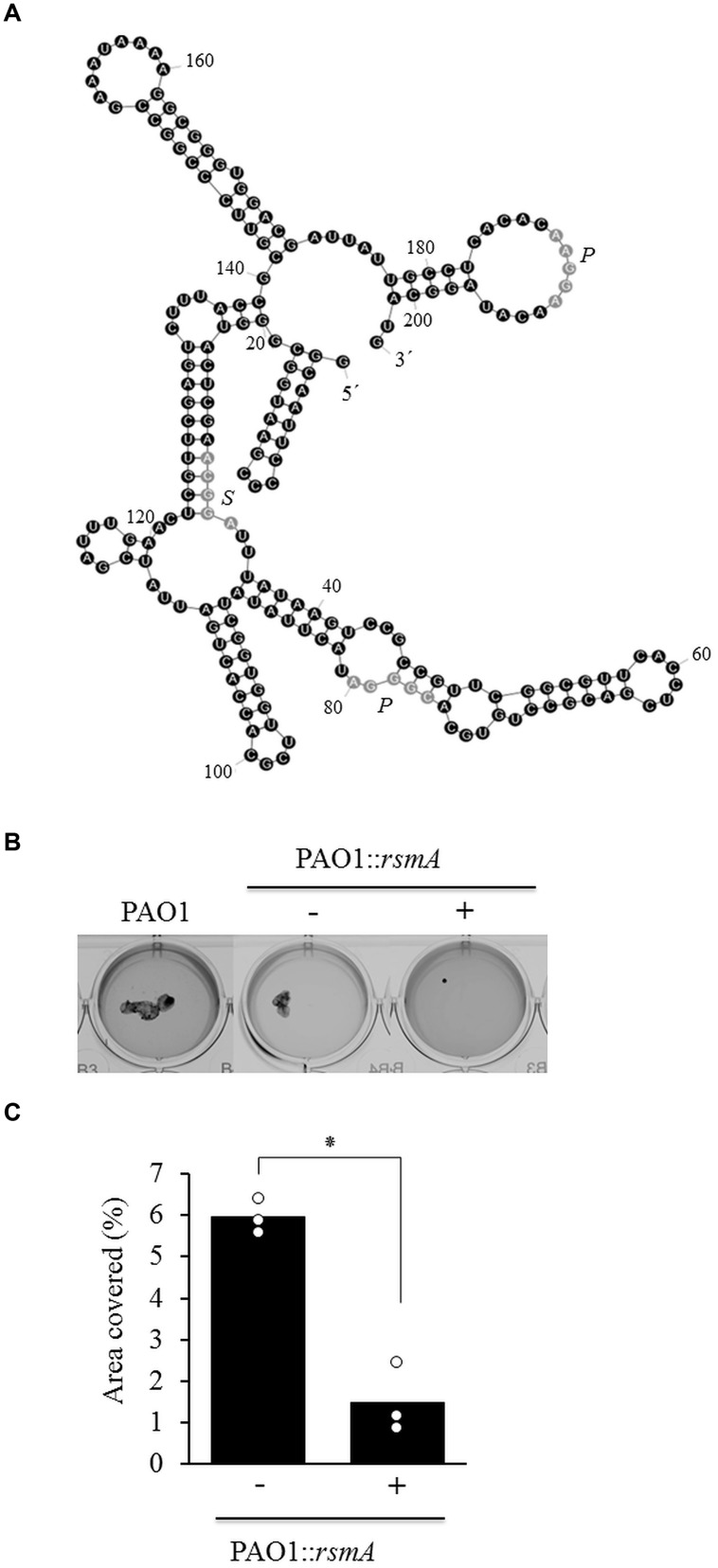
**(A)** Structural prediction of the *cupA1* mRNA leader sequence using mFold (Zuker, 2003) with predicted primary (*P*) and secondary (*S*) RsmA binding sites. **(B)** Phenotypic analysis of strains PAO1 and PAO1::*rsmA* and the impact of RsmA induction (+) on macroscopic cell aggregation during growth with SDS. **(C)** Quantitative analysis of the percentage covered by macroscopic aggregates of different strains. Bars represent the mean value from three individual experiments and are accompanied by the individual data points. Statistical significance is indicated (^∗^*p* = 0.001).

Surprisingly, no differences in *cupA1* transcription could be observed between the different strains and growth conditions tested. The fact that the increased *cupA1* mRNA levels detected in earlier studies did not correlate with increased activation of the transcriptional *cupA1* reporter under identical conditions could be indicative of a posttranscriptional regulatory mechanism. The RsmA/*rsmY*/*Z* system has been shown to play an important role in the posttranscriptional regulation of many genes in *P. aeruginosa*, including those involved in biofilm formation (Burrowes et al., 2006; Brencic and Lory, 2009). Analysis of the *cupA1* leader sequence revealed a potential primary RsmA binding site that shared 90% sequence identity with the known SELEX binding consensus (ACAA**GGA**AcA; sequence divergence is indicated by lowercase), including the GGA core motif (bold and underlined) located 6–15 nucleotides upstream from the *cupA1* start codon (**Figure 3A**; positions 186–195). In addition, one primary (29–33) and one secondary binding site (76–80) were identified as defined by a recent computational approach (Kulkarni et al., 2014). This sequence was then further analyzed by structural prediction using the mFold web server (Zuker, 2003). The structural prediction with the highest stability was found to harbor multiple stem–loop structures, and notably the SELEX derived RsmA consensus sequence is predicted to be located at the end of a stem–loop (nucleotides 186–195) which overlaps with the predicted SD sequence for *cupA1* (nucleotides 188–193), which is predicted to be the ideal RsmA binding conditions.

To explore the potential influence of RsmA on macroscopic cell aggregation during growth with SDS, we used a strain harboring an arabinose inducible copy of *rsmA* at a neutral site of the *P. aeruginosa* chromosome. The induction of RsmA completely abolished macroscopic aggregate formation during growth with SDS compared to the non-induced control (**Figure 3B**). Quantitative analysis of these strains revealed that the induction of *rsmA* caused a fourfold reduction in the area covered by macroscopic aggregates when compared to the non-induced control during growth with SDS (**Figure 3C**).

### RsmA Overexpression Reduces *cupA1* mRNA Stability

**FIGURE 4:**
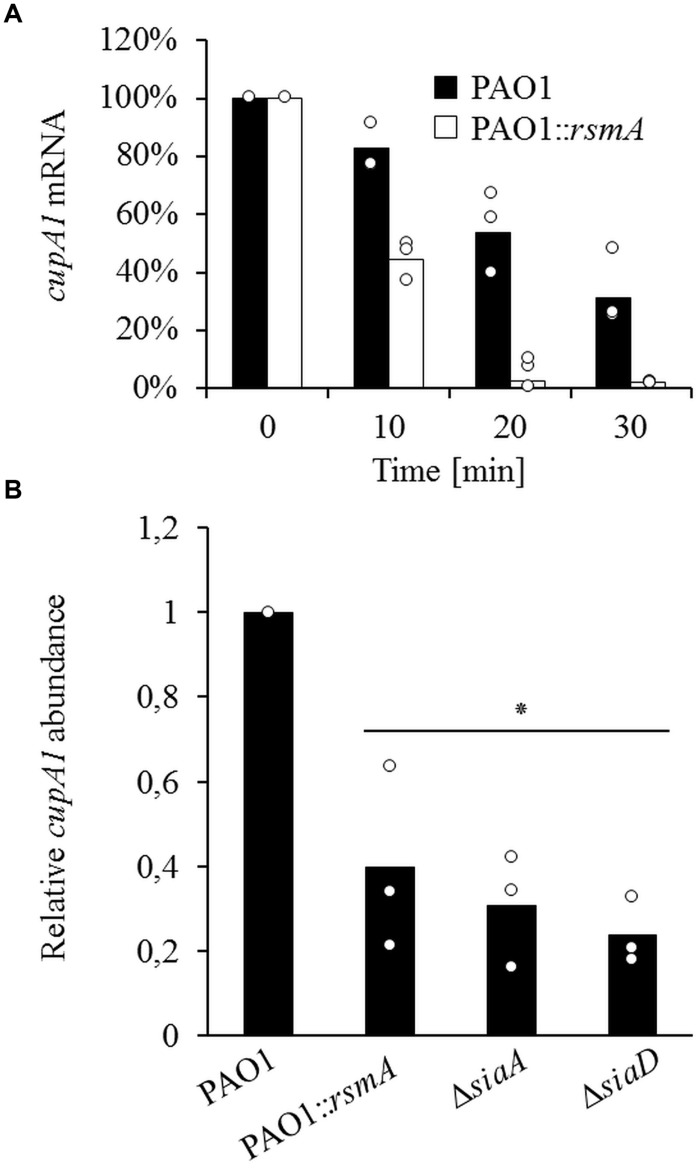
**(A)** The effect of RsmA overexpression on the stability of *cupA* mRNA as determined by qRT-PCR. Data were normalized by the use of equivalent mRNA and analyzed by the 2^-ΔCt^ method. **(B)** Relative abundance of *cupA1* mRNA during growth in SDS for strains PAO1, PAO1::*rsmA* and the mutant strains Δ*siaA* and Δ*siaD*. Data were normalized to the *proC* housekeeping gene by the 2^-ΔΔCt^ method. Bars represent the mean value from three individual experiments and are accompanied by the individual data points. Statistical significance compared to PAO1 is indicated (^∗^*p* ≤ 0.01).

To investigate whether RsmA influences *cupA1* mRNA stability, we analyzed the mRNA decay of *cupA1* by qRT-PCR and found that the overproduction of RsmA strongly reduced the stability of the *cupA1* transcript for all time points tested (**Figure 4A**). Compared to cells of the wild-type strain, which retained 83, 54, and 31% of the initial *cupA1* transcript levels after 10, 20, and 30 min respectively, cells containing increased levels of RsmA showed a much faster rate of decay with only 43, 2, and 2% of the initially detected transcript levels present at the same time points. In addition, quantification of the relative abundance of the *cupA1* transcript revealed that *cupA1* levels were reduced during growth with SDS when RsmA was overproduced compared to the parental strain (0.35 fold) (**Figure 4B**). Further, the non-aggregative Δ*siaA* and Δ*siaD* mutant strains also displayed reduced *cupA1* mRNA levels (0.46 fold and 0.23 fold, respectively) when compared to wild-type cells.

### RsmA Directly Binds to *cupA1* mRNA

**FIGURE 5:**
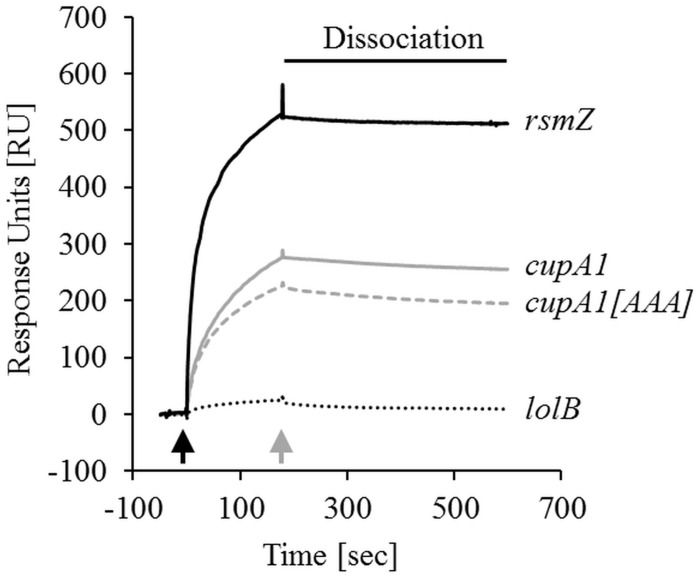
**Protein-RNA binding studies.** Surface plasmon resonance with purified RsmA-His6 protein (961 RU ± 18.5 RU) immobilized on an NTA sensor chip (GE Healthcare) and 200 nM of the *in vitro* generated RNAs as interaction partners, namely the *cupA1* RNA, the *cupA1[AAA]* RNA harboring a GGA to AAA mutation of the RsmA binding site overlapping with the SD sequence, the positive control RNA *rsmZ* and the negative control RNA *lolB*. The response units (RU), which indicate direct binding to RsmA, were normalized to the molecular weight of the *rsmZ* RNA. The start of sample injection (*black arrow*) and the end of sample injection (*gray arrow*) are indicated. Detailed information about the experimental setup and data analysis can be found in the Supplementary Material.

In order to test if RsmA regulates *cupA1* stability by directly binding to its mRNA, we performed surface plasmon resonance measurements. For these experiments, 961 (±18.5) response units (RU) of purified RsmA-His6 protein was immobilized on a Ni-NTA sensor chip, which reliably allowed the binding analysis of different *in vivo* generated RNA molecules in independent runs. These experiments revealed that samples with 50, 100, and 200 nM of RNA comprised of the 200 nt upstream of the *cupA1* starting codon resulted in a specific increase in RU over time (**Figure 5**; Supplementary Figures S1A,B). In contrast, the negative control RNA *lolB* showed no specific increase in RU at 200 nM, demonstrating a specific interaction of the *cupA1* RNA with the immobilized RsmA protein. Compared to RNA of the known RsmA antagonist *rsmZ*, the *cupA1* RNA displayed 67, 59, and 50% lower RU at concentrations of 50, 100, and 200 nM (determined at the end of the dissociation) when normalized to molecular weight (**Figure 5**; Supplementary Figures S1A,B). In addition, mutation of the core motif of the potential RsmA binding site overlapping with the SD sequence of *cupA1* (GGA to AAA mutation; *cupA1[AAA]*) resulted in 28, 31, and 24% lower RU compared to the same concentrations of the native *cupA1* RNA. Thus, RsmA appears to bind the *cupA1* mRNA leader sequence and the GGA motif at the SD sequence is involved in this binding, which would be consistent with the reduced mRNA stability observed when RsmA was overexpressed in the presence of SDS.

### SiaA/D are Required for *rsmZ* Expression and Increased Intracellular c-di-GMP Levels During Growth with SDS

**FIGURE 6:**
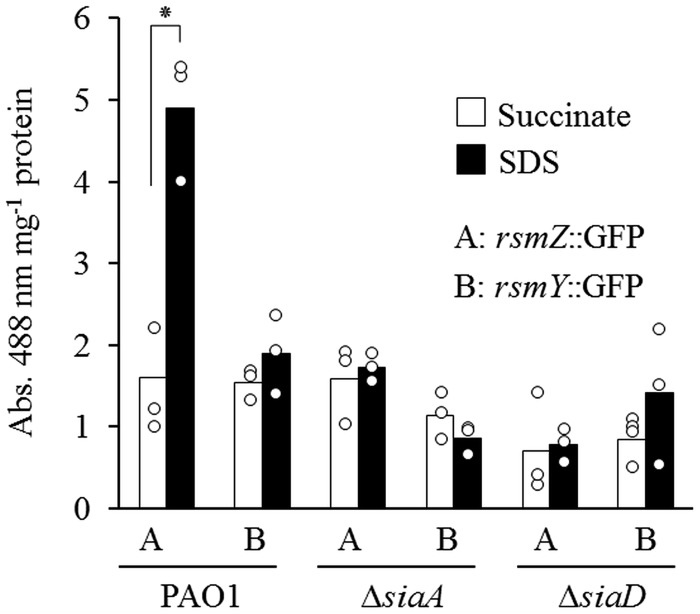
**(A)** Phenotypic analysis of *rsmY* and *rsmZ* overexpression in the non-aggregative Δ*siaA* and Δ*siaD* mutants during growth with 3.5 mM SDS. **(B)** Transcriptional reporter analysis of *rsmZ* and *rsmY* for strain PAO1 and the Δ*siaA* and Δ*siaD* mutant strains. Data is expressed as the signal intensity at 488 nm normalized to the total protein of the sample (Abs. 488 nm mg^-1^ protein). Bars represent the mean value from three individual experiments and are accompanied by the individual data points. Statistical significance is indicated (^∗^*p* = 0.004).

The ability of RsmA to negatively influence macroscopic aggregate formation during growth with SDS and its impact on the stability of the *cupA1* mRNA transcript raised the question of whether SiaA/D could influence RsmA activity. As such, we investigated the transcriptional activation of the known RsmA antagonists *rsmY/Z* during growth with SDS. Experiments with previously described *rsmY* and *rsmZ* transcriptional reporters (Chua et al., 2014) found that the transcription of *rsmZ* was increased threefold for wild-type cells grown with SDS when compared to those grown with succinate, whereas the transcription of *rsmY* was not altered (**Figure 6**). This increase in *rsmZ* transcription was dependent on both SiaA and SiaD, as no increase was observed for either of the Δ*siaA* or Δ*siaD* mutant strains.

**FIGURE 7:**
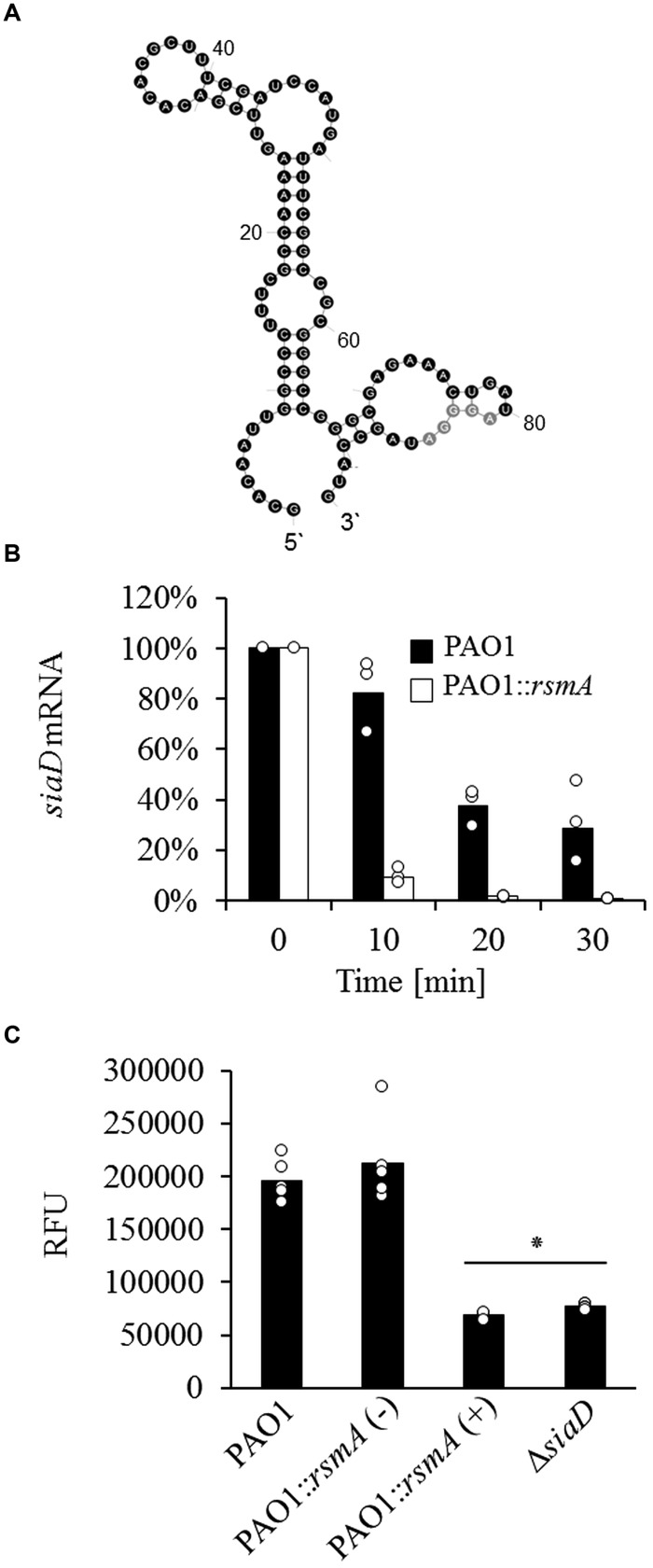
**(A)** Structural prediction of the *siaABCD* leader sequence using mFold (Zuker, 2003) with the predicted primary RsmA binding site (*gray*) **(B)** Impact of RsmA overexpression on the stability of *siaD* mRNA as determined by qRT-PCR. Data were normalized by the use of equivalent mRNA and analyzed by the 2^-ΔCt^ method. **(C)** c-di-GMP levels of cells quantified as relative fluorescence units (RFU with excitation/emission of 485/535 nm) normalized to optical density by using the pCdrA::*gfp^c^* reporter for the wild-type, PAO1::*rsmA* [with (+) and without (-) RsmA induction (2% arabinose)], and the Δ*siaD* strains. Bars represent the mean value from three **(B)** or six **(C)** individual experiments and are accompanied by the individual data points. Statistical significance compared to strain PAO1 and PAO1::*rsmA* is indicated (^∗^*p* = 0.001).

It has been previously shown that the RsmA/*rsmY*/*Z* system influences DGC expression in *P. aeruginosa* (Chua et al., 2014; Moscoso et al., 2014; Bouffartigues et al., 2015). Given that RsmA and SiaD inversely regulate macroscopic aggregate formation during growth with SDS, we speculated that this may involve a regulatory feedback where RsmA negatively affects the expression of *siaD*. The *siaD* gene is transcribed in an operon together with *siaA*, *siaB* (PA0171) and *siaC* (PA0170) (Klebensberger et al., 2009), so the 90 nt leader sequence of *siaA* from the previously reported transcriptional start site (Jones et al., 2014) to the translational start codon was analyzed for RsmA binding sites. Indeed, a potential primary RsmA binding site with 70% similarity to the SELEX sequence (AuAG**GGA**Uag, sequence divergence is indicated in lowercase), including the core GGA motif (bold and underlined), was found 3–12 nucleotides upstream of the start codon of *siaA* (**Figure 7A**). Structural prediction of this sequence using mFold placed the potential binding site as part of a stem–loop structure (nucleotides 67–90) overlapping with the SD sequence of *siaA*, suggesting that RsmA could be involved in the regulation of *siaABCD* mRNA stability. In order to test this hypothesis, we quantified the mRNA decay of *siaD*. While the wild-type strain retained 82, 32, and 28% of the initial *siaD* transcript levels after 10, 20, and 30 min, the RsmA overproducing strain showed a much faster rate of decay with only 9, 2, and 1% of the initially detected transcript levels being detected at these same time points (**Figure 7B**).

The ability for RsmA overproduction to reduce the stability of the mRNA encoding the DGC SiaD suggested that it could negatively impact the levels of c-di-GMP within the cell during growth with SDS. As such, we quantified the intracellular levels of c-di-GMP with the previously described c-di-GMP responsive CdrA*::gfp*^C^ reporter construct for the wild-type strain, the RsmA overproducing strain and the Δ*siaD* mutant during growth with SDS (Rybtke et al., 2012). While the non-induced control retained wild-type levels of c-di-GMP, the overproduction of RsmA reduced the intracellular levels of c-di-GMP to just 35.3% of those present for cells of the wild-type strain during growth with SDS (**Figure 7C**). Notably, a similar reduction in c-di-GMP levels was found for the Δ*siaD* strain, further supporting the link between SiaA/D dependent c-di-GMP biosynthesis, the connection with RsmA and macroscopic cell aggregation.

## Discussion

We have previously described that macroscopic aggregate formation of *P. aeruginosa* during growth with, or in the presence of the surfactant SDS (Klebensberger et al., 2006). We proposed that activation of the sensor protein and putative ser/thr phosphatase SiaA and the regulation of c-di-GMP levels by SiaD modulate this phenotype (Klebensberger et al., 2007, 2009). The functional role of SiaD in c-di-GMP biosynthesis is based on several lines of evidence. Firstly, loss of *siaD* or the depletion of c-di-GMP eliminates macroscopic aggregate formation (Klebensberger et al., 2009). Secondly, SiaD was found to be essential for purified Psl polysaccharide to stimulate increased c-di-GMP levels, in addition to the cyclase SadC (Irie et al., 2012). Thirdly, mutation of the active site responsible for c-di-GMP catalysis produces a *siaD* allele that is unable to complement the Δ*siaD* mutant (this study). Fourthly, mutation of *siaD* strongly reduced the activity of the pCdrA::*gfp*^C^ c-di-GMP responsive reporter (this study). Lastly, growth in the presence of stress-inducing tellurite was found to increase c-di-GMP levels in a SiaD dependent manner (Chua et al., 2015). The lack of c-di-GMP catalysis previously reported for purified SiaD protein (Kulesekara et al., 2006) together with the fact that the overexpression of SiaD does not complement a Δ*siaA* mutant (Klebensberger et al., 2009) suggests that functional activation of SiaD is dependent on SiaA during cell stress (e.g., growth with SDS or exposure to tellurite). In addition to the predicted biosynthesis of c-di-GMP, our data further indicate a functional regulatory role for the I-site in SiaD. This is based on the observation that mutation of the I-site in SiaD produced an allele that increased macroscopic aggregation greater than the native *siaD* allele. This motif is well known for binding c-di-GMP and thereby limiting further activity of the DGCs in a negative feedback loop (Christen et al., 2006). From our data, we suggest that this is also a plausible explanation for its function in SiaD.

### Regulation of *cupA*

At least three cell surface adhesins are essential for macroscopic cell aggregation during growth with, or in the presence of the surfactant SDS, and are targets for the SiaA/D pathway; the CdrB proteinaceous adhesin, the Psl polysaccharide and the CupA fimbriae (Klebensberger et al., 2009). Each of these adhesins has also been shown to play an important role in promoting surface attached biofilm formation (Vallet et al., 2001; Jackson et al., 2004; Borlee et al., 2010). In contrast to Psl and CdrAB (Burrowes et al., 2006; Brencic and Lory, 2009; Borlee et al., 2010; Irie et al., 2012), the evidence that c-di-GMP regulates CupA fimbriae is conflicting (Meissner et al., 2007; Starkey et al., 2009). Further, no role for RsmA has been suggested. In this study, we present compelling evidence that the *cupA1* leader is a novel, direct target for RsmA, and show that RsmA overproduction results in loss of macroscopic aggregation during growth with SDS. Sequence analysis and structural prediction first identified several potential RsmA binding sites in the *cupA1* leader. Most strikingly, one of the sites is predicted to be exposed in single stranded RNA at the end of a stem loop structure overlapping the SD sequence of *cupA1*, which represents the most common feature of mRNA targets directly regulated by RsmA (Timmermans and Van Melderen, 2010; Vakulskas et al., 2015). Subsequent surface plasmon resonance analysis demonstrated that *in vitro* generated *cupA1* RNA specifically binds to purified RsmA protein. The comparison of relative binding affinities for different RsmA-RNA interactions demonstrated that *rsmZ* exhibits ∼2 fold stronger affinity to RsmA than that of the *cupA1* RNA. Interestingly, mutation of the GGA core motif of the predicted primary binding site overlapping the SD sequence of *cupA1* reduced RsmA binding, although an interaction with lower affinity was still detected that displayed stable binding over time. This could be explained by the presence of additional RsmA binding sites within the *cupA1* RNA molecule (Babitzke and Romeo, 2007; Timmermans and Van Melderen, 2010; Romeo et al., 2013). The RsmA homolog CsrA functions as a homodimer that is able to bind to two locations of target mRNA that are separated by 10–63 nt (Mercante et al., 2009). The predicted additional primary RsmA binding site and the experimentally defined RsmA target in the *cupA1* leader are separated by 14 nt, a distance similar to the 18 nt separation that has been described as the optimal bridging distance (Mercante et al., 2009). It has also been hypothesized that RsmA could function by a sequential binding mechanism, where an initial binding event enables the second binding surface present in the homodimer to access the mRNA (Mercante et al., 2009; Lapouge et al., 2013). The binding of multiple sites within target mRNA is known to be physiologically relevant for regulating target gene expression for CsrA (Mercante et al., 2009). While further experiments would be required to prove that the additional binding sites in the *cupA1* leader are indeed functional, this study clearly shows that RsmA specifically binds the *cupA1* transcript in a manner that is partially dependent on the primary binding site overlapping its SD sequence. Given that RsmA has been well characterized for its ability to impede ribosome access when binding to mRNA overlapping a SD sequence, resulting in translational repression and increased mRNA turnover (Romeo et al., 2013), our results suggest a similar mechanism for the regulation of *cupA1* mRNA.

Notably, a recent sequence based informatics approach did not identify the *cupA1* gene as a potential target for RsmA regulation (Kulkarni et al., 2014). However, this can be explained by the high levels of stringency required to minimize the number of false positive sequences, which is problematic because of the similarity between the RsmA consensus and the widespread SD sequence (Kulkarni et al., 2014). While the 200 nt upstream of *cupA1* contains two primary and one secondary potential binding sites for RsmA, the informatics algorithm used in the aforementioned study required either three primary, or two primary and two secondary sequence sites for an mRNA leader to be subjected to further analysis (Kulkarni et al., 2014). In addition, earlier experimental approaches using Δ*rsmA* mutants also did not identify genes of the *cupA* operon as part of the RsmA regulon (Burrowes et al., 2006; Brencic and Lory, 2009). In this regard, it is important to note that these experiments were performed in rich LB medium, a growth condition in which the transcription of *cupA1* is known to be strongly repressed by the global repressor MvaT, and to a lesser degree by MvaU (Vallet et al., 2004; Vallet-Gely et al., 2005, 2007). In addition, we show that the CupA fimbriae are essential for macroscopic aggregation during growth with SDS. As such, it seems possible that transcriptomic studies comparing wild-type cells with those of a Δ*rsmA* mutant would fail to identify the influence of RsmA on *cupA1* regulation due to the use of rich medium for growth, and the low levels of *cupA1* transcription in the absence of the signal generated by SDS.

The global regulator RsmA and the second messenger c-di-GMP have been shown to inversely regulate various phenotypic traits in *P. aeruginosa* (Frangipani et al., 2014; Moscoso et al., 2014; Chen et al., 2015; Petrova et al., 2015). Accordingly, each of these regulators is influenced by a large range of different sensory inputs. Here, we show that the SiaA/D signal transduction module regulates phenotype expression in a c-di-GMP- and RsmA dependent manner. These results are in accordance with the recent finding that SiaD influences the production of RsmA regulated Psl polysaccharide at the posttranscriptional level (Irie et al., 2012). Our present study demonstrates that the connection between SiaD and RsmA involves the presence of a functional SiaA protein and the transcriptional regulation of *rsmZ.* This is in line with the observation that SiaD dependent pyoverdine expression in cells with artificially increased c-di-GMP levels involves the expression of *rsmY*/*Z* (Chen et al., 2015). While the specific mechanism by which SiaA/D impacts the transcription of *rsmZ* remains unknown, our data and the study of Chen et al. (2015) indicate that it involves the functional activation of SiaD and as a consequent the modulation of c-di-GMP levels. Finally, we provide strong evidence that the *siaABCD* operon is also under regulatory control of the RsmA/*rsmY*/*Z* system. The *siaABCD* operon contains a potential RsmA binding site overlapping with the SD sequence, and the decay of *siaD* was strongly dependent on RsmA induction. This is supported by earlier microarray studies comparing a Δ*rsmA* and a Δ*retS* mutant (a hybrid sensor kinase and response regulator that regulates *rsmZ* to inhibit RsmA) with their respective parental controls, which identified a significant up-regulation of *siaC* and the complete *siaABCD* operon, respectively (Goodman et al., 2004; Brencic and Lory, 2009). As further support, we demonstrate that overexpression of RsmA results in a similar decrease in intracellular c-di-GMP levels during growth with SDS as the deletion of *siaD*, providing strong evidence of a novel interconnection between these two global regulatory networks.

**FIGURE 8:**
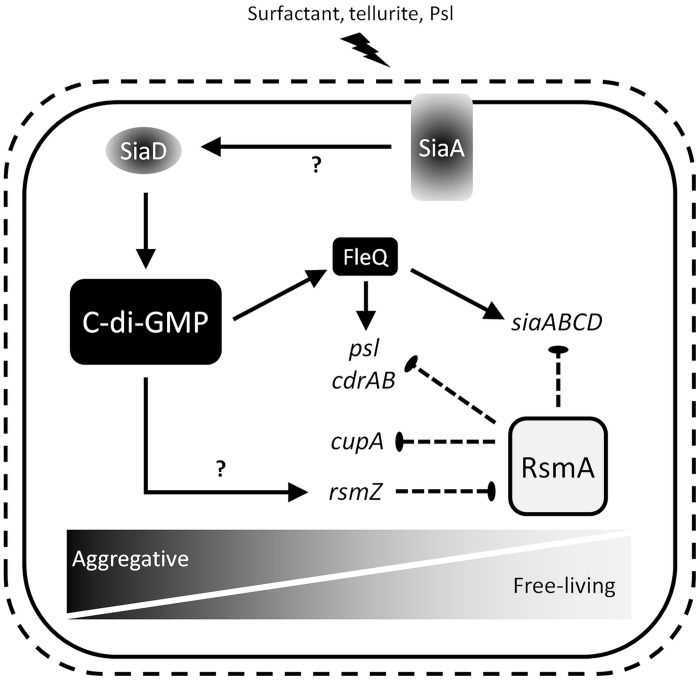
**Working hypothesis for SiaA/D function.** Detailed information is given in the text.

From these data, we propose a working hypothesis (**Figure 8**) in which SiaA/D coordinates the antagonistic activities of c-di-GMP and RsmA to control cellular aggregation in response to multiple environmental conditions, including the presence of SDS and tellurite during growth, and the signaling properties of the matrix component Psl (Irie et al., 2012; Chua et al., 2015). In the absence of surfactant stress, the DGC SiaD is inactive and thus RsmA is not repressed by the c-di-GMP dependent transcription of *rsmZ*, resulting in a shift toward a planktonic, non-aggregative phenotype. In the presence of surfactant stress, the activation of SiaD by SiaA influences the expression of target genes by c-di-GMP biosynthesis and the transcriptional activation of *rsmZ*. The exact mechanisms of SiaD and *rsmZ* activation remain incompletely understood, and are under current investigation in our laboratory. Notably, both RsmA, and c-di-GMP *via* the transcriptional regulator FleQ (Baraquet and Harwood, 2015), are also proposed to exhibit a regulatory feedback on the *siaABCD* operon. This complex and unique regulatory feedback circuit by which SiaA/D seems to be interconnected to c-di-GMP and RsmA signaling likely reflects the importance of appropriately regulating cellular aggregation, and could allow for a rapid and energy-efficient adaptation in response to external stimuli. This is of particular interest as both RsmA and c-di-GMP regulate phenotypic traits which are of clinical relevance, such as stress tolerance, acute virulence, and chronic persistence.

## Author Contributions

BC, SR, SK, and JK designed research. BC, VD, MC, and JK performed experiments. All authors analyzed data. BC and JK wrote the paper with contributions of VD, SR, and SK during revision.

## Conflict of Interest Statement

The authors declare that the research was conducted in the absence of any commercial or financial relationships that could be construed as a potential conflict of interest. The reviewer MS and handling Editor declared a current collaboration and the handling Editor states that the process nevertheless met the standards of a fair and objective review.
