# Supplementary material for: SiaA/D Interconnects c-di-GMP and RsmA Signaling to Coordinate Cellular Aggregation of Pseudomonas aeruginosa in Response to Environmental Conditions
Source: Front Microbiol. 2016 Feb 26;7:179. doi: 10.3389/fmicb.2016.00179 (PMC4768041; doi:10.3389/fmicb.2016.00179)
Supplement: Supplementary file 1 [file Data_Sheet_1.PDF]

Supplementary Material

SiaA/D interconnects c-di-GMP and RsmA signaling to coordinate cellular aggregation of *Pseudomonas aeruginosa* in response to environmental conditions

Brendan Colley, Verena Dederer, Michael Carnell, Staffan Kjelleberg, Scott A. Rice, and Janosch Klebensberger*

*** Correspondence:** Corresponding Author: janosch.klebensberger@itb.uni-stuttgart.de

1 Supplementary Data

Plasmid construction

The PCR products pUC19[*lolB*] and pUC19[*rsmZ*] were digested with EcoRI and BamHI and ligated into the corresponding vector linearized with the same restriction enzymes. For construction of pBAD33[RsmA-His6], the previously constructed vector pBAD33[*R*-IRED-*Sr*] was digested with NdeI and HindIII and dephosphorylated using alkaline phosphatase (Fermentas) according to the manufacturer's instructions. The PCR fragment containing the C-terminal His6 tagged *rsmA* gene was digested with the same restriction enzymes, ligated into pBAD33, and finally transformed into the *E. coli* strain BW25113.

Site directed mutagenesis was used to generate the pBBR[*siaD*^{R130A}], pBBR[*siaD*^{G140A}] and pUC18[*cupA1*_AAA] plasmids. The corresponding mutations were introduced with the self-complementary PCR primers SiaD_A_F, SiaD_A_R, CupA_AAA_F and CupA_AAA_R (Table S2) by PCR using pBBR[*siaD*] and pUC18[*cupA1*] (kindly provided by Tina Jaeger from the Biozentrum, Basel) as DNA templates and a polymerase with proof reading activity. Subsequently, the PCR product was digested with DpnI, purified (DNA Clean and Concentrator Kit; Zymo Research), and transformed into competent cells of DH5α. Correct construction of all plasmids was verified by DNA sequencing.

In vitro *transcription and RNA purification*

For generating RNA molecules, the DNA templates pUC18[*cupA1*], pUC18[*cupA1*_AAA], pUC18[*rsmZ*] and pUC18[*lolB*] were digested with BamHI and EcoRI, separated by gel electrophoresis, and the insert of the plasmids was purified (Clean Gel DNA Recovery Kit; Zymo Research) and used as a template for a PCR with proof-reading polymerases with the primers used for cloning of the DNA templates (Table S2). After purification of the PCR product, *in vitro* transcription was performed with 1 µg template DNA, 1x transcription buffer, 2 mM rNTP mix, 50 U RiboLock RNase inhibitor (Thermo Scientific) and 30 U T7 RNA polymerase (Thermo Scientific) with incubation at 37°C for 2 h. After incubation, 2 U DNase I and 6 mM $MgCl_2$ was added to remove DNA. After heat inactivation of the DNase for 10 min at 80°C, the RNA fragments were

purified using the RNA Clean and Concentrator 5 kit (Zymo Research) and stored in nuclease free water at -80°C.

Protein production, purification and concentration

For RsmA-His6 production, overnight cultures of strain BW25113 harboring pBAD33[RsmA-His6] in TB (12 g L^{-1} tryptone, 24 g L^{-1} yeast extract, 2.2 g L^{-1} KH_2PO_4, 9.4 g L^{-1} K_2HPO_4, 4 mL L^{-1} glycerol) supplemented with 30 µg mL^{-1} chloramphenicol were used to inoculate 2 × 250 mL fresh TB medium in 2 L baffled Erlenmeyer flasks and incubated at 30°C with shaking at 180 rpm (Multitron, Infors HT AG). After the cultures reached an OD_{600} = 0.8, L-Arabinose was added (0.2% w / v) to induce protein production. After 3 h of incubation, cells were chilled on ice for 10 min with regular mixing and harvested by centrifugation at 4°C and 9,500 × *g* for 10 min (Eppendorf 5810 R), re-suspended in 20 mL buffer solution containing 50 mM Tris HCL, 150 mM NaCl, 1% glycerol (v/v), 1 µg mL^{-1} DnaseI, 1 µg mL^{-1} Lysozyme, 1 mM PMSF at pH: 7.5, homogenized with a tissue grinder and lysed using an Emulsiflex-C5 (Avestin). Cell debris were separated by centrifugation at 9,500 × *g*, the supernatant was transferred to fresh tubes, and proteins were purified using two His GraviTrap TALON columns (GE Healthcare). The columns were equilibrated using 10 mL cold equilibration buffer (50 mM Tris-HCL, 150 mM NaCL, 1% glycerol (v/v) with 5 mM imidazole at pH: 8), and then 2 × 10 mL of the cell lysates were used to load the columns. The columns were then washed once with 5 mL equilibration buffer containing 5 mM imidazole and twice with equilibration buffer containing 30 mM imidazole. Finally, RsmA-His6 was eluted from both columns with a total volume of 4 ml equilibration buffer containing 200 mM imidazole.

The protein sample was dialyzed using a Float-A-Lyzer G2 device (Spectrum Laboratories) against 800 mL (overnight at 4°C) and 200 mL (4 h at RT) equilibration buffer without imidazole. Subsequently, 2 × 400 µL protein samples were concentrated using an Amicon Ultra-0.5 Centrifugal Filter Device (3 kDa cut-off) and protein concentrations of the samples were measured using a Pierce BCA Protein Assay kit according to the manufacturer’s instructions (Pierce).

Surface plasmon resonance measurements and data analysis

Protein-RNA interactions were measured as the difference in response units (RU) using surface plasmon resonance with a BIACore3000 system equipped with a NTA sensor chip (GE Healthcare) for capturing His-tagged proteins.

Prior to the measurements, solutions were filter sterilized (0.2 µM, VWR International) and degassed for 10 min at 30°C and the purified RsmA-His6 protein was diluted to a final concentration of 2 µg mL^{-1} in running buffer (10 mM Tris-HCL at pH: 7.5, 150 mM NaCL, 0.05% Tween (v/v), 1 mM imidazol, 5 ng mL^{-1} total yeast RNA (Ambion)). In addition, different concentrations of the RNA samples were prepared in RNAse-free vials (200 nM, 100 nM, 50 nM) in running buffer immediately before starting a measurement. For each experiment the interaction flow-cell (Fc2) was subjected to the following protocol cycle with a flow rate of 20 µL min^{-1}:

1) 200 mM imidazole solution for 1 min following a 1 min equilibration time; 2) 1.5 M NaCl solution containing 350 mM EDTA for 1 min; 3) 500 µM $NiCl_2$ solution for 1 min; 4) 3 mM EDTA solution for 1 min; 5) Running buffer for 1 min; 6) RsmA-His6 solution for 1 min followed by a 3 min equilibration time; 7) Sample injection for 2 min followed by a 6 min dissociation time; 8) 200 mM imidazole solution for 1 min. Further, a reference flow-cell (Fc1) was monitored which was identically treated as described above, with the exception of the $NiCl_2$ activation of the NTA surface (step 3).

With the described protocol, consistent levels of RsmA immobilization (961 RU ± 18,5 RU) and complete regeneration of the NTA surface were achieved. For analysis, all data were referenced by subtracting the signal from the reference flow-cell (Fc2-Fc1). In addition, a blank run subtraction (buffer injection sample) was used for all RNA containing samples using the Y-transform function of the BIAEvaluation software (Version: 4.1). For direct comparison of different samples, the corresponding sensograms were loaded into the BIAEvaluation software, the sample injection start was defined manually (X-transform function) and a baseline was generated by averaging 10 sec of data 5 sec before sample injection (Y-Transform function). Finally, all data 50 sec prior to sample injection and 10 sec prior to the end of the dissociation time was excluded from the sensogram. For generating graphs, these datasets were exported as a text file and imported into Microsoft Excel.

As the change in RU is proportional to the molecular weight of the corresponding interaction partner, the datasets were normalised to the molecular weight of the *rsmZ* RNA in order to allow comparison between different RNAs. The molecular weight of each RNA fragment was calculated using the online Oligonucleotide Properties Calculator (http://www.basic.northwestern.edu/biotools/OligoCalc.html) and the complete Excel dataset was corrected accordingly (see Table S3). Each sample was measured at least twice on different days leading to similar results.

2 Supplementary Figures and Tables

2.1 Supplementary Figures

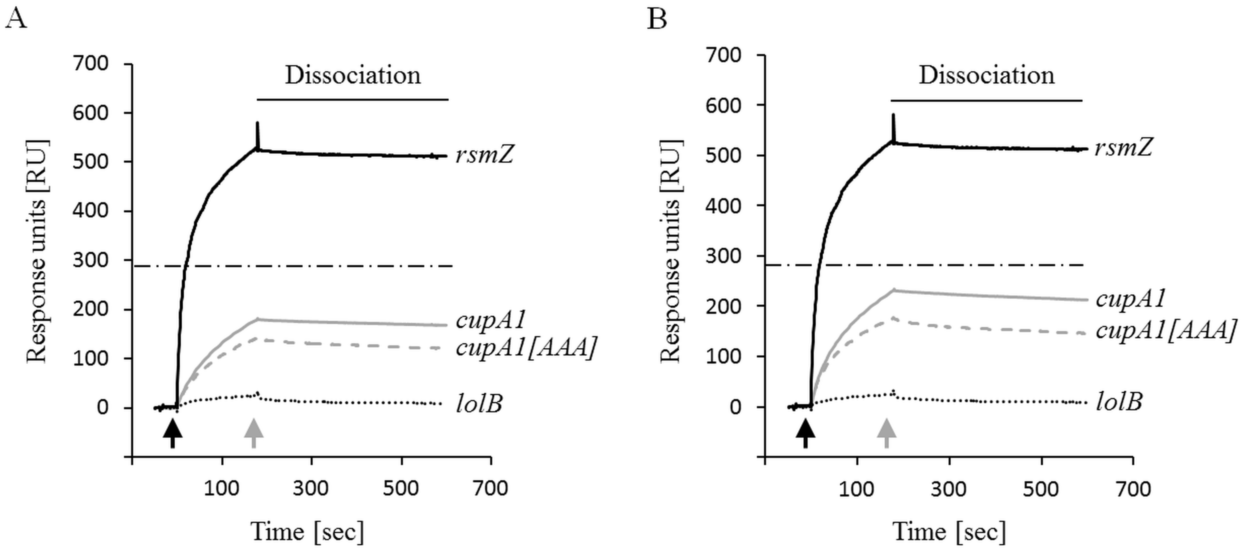

Supplementary Figure S1. Protein-RNA binding studies using surface plasmon resonance with purified RsmA-His6 protein (961 RU ± 18,5 RU) immobilised on an NTA sensor chip (GE Healthcare) and 50 nM (A) or 100 nM (B) of *in vitro* generated *cupA1* or *cupA1[AAA]* RNAs as interaction partners. The negative- (*lolB*) and positive (*rsmZ*) control RNAs were used at a concentration of 200 nM. The response units (RU) that indicate direct binding to RsmA were normalized to the molecular weight of *rsmZ* RNA. The start of sample injection (black arrow) and

the end of sample injection (grey arrow) are indicated. The maximum RU obtained after injection of 200 nM (see Figure 5) of *cupA1* RNA is indicated (dash-dotted black line).

2.2 Supplementary Tables

Supplementary Table S1. Strains and plasmids used in the study.

**Strains**	**Relevant features**	**Reference**
P. aeruginosa		
PAO1	Wild-type *Pseudomonas aeruginosa*	Holloway Collection
Δ*siaA*	PAO1 with a 6 bp deletion in *siaA*	(Klebensberger *et al.*, 2009)
Δ*siaD*	PAO1 with a markerless *res*-site insertion in *siaD* (KO0169)	(Klebensberger *et al.*, 2009)
PAO1::*rsmA*	PAO1 with *rsmA* allele under control of the arabinose inducible P_{BAD} promoter at the *att*Tn7 site	This study
Δ*cupA3*	PAO1 with non-polar *cupA3* deletion	This study
Δ*psl*	PAO1 with non-polar *pslBCDE* deletion	This study
MPAO1_KO*cupA3*	MPAO1 with Gm^r *cupA3* insertion and flanking FRT sites, source of Δ*cupA*	(Starkey *et al.*, 2009)
MPAO1_KO*pslBCD*	MPAO1 with Gm^r *pslBCDE* insertion and flanking FRT sites, source of Δ*psl*	(Kirisits *et al.*, 2005)
E. coli		
DH5α	*fhuA2 lac(del)U169 phoA glnV44 Φ80' lacZ(del)M15 gyrA96 recA1 relA1 endA1 thi-1 hsdR17*	(Bethesda, 1986)
BW25113	F^- *Δ(araD-araB)567 ΔlacZ4787(::rrnB-*	(Baba *et al.*, 2006)

	3), rph-1 Δ(rhaD-rhaB)568 hsdR514 λ^{-}	
**Plasmids**		
TopoKO0169	pCR2.1 harbouring the *siaD* gene	(Klebensberger *et al.*, 2009)
pBBR	Broad host range expression plasmid pBBR1MCS-5, Gm^{r}	(Kovach *et al.*, 1995)
pJN105	Arabinose inducible (*araC*-P_{BAD}) plasmid based on pBBR1MCS-5	(Newman and Fuqua, 1999)
pUC18	IPTG inducible cloning vector, Ap^{r}	Thermo Scientific
pUC19	IPTG inducible cloning vector, Ap^{r}	Thermo Scientific
pBAD33<br><br>[*R*-IRED-*Sr*]	Arabinose inducible plasmid harboring an optimized ribosomal binding site and the synthetic gene encoding an imine reductase (*R*-IRED) from *Streptosporangium roseum* as a NdeI – HindIII fragment , Cm^{r}	(Scheller *et al.*, 2014)
pBAD33<br><br>[RsmA-His6]	BAD33 harbouring the *rsmA* gene from *P. aeuruginosa* as a NdeI – HindIII fragment fused to a C-terminal His6 tag derived from pBAD33[*R*-IRED-*Sr*]	This study
pBBR[*siaD*]	pBBR1MCS-5 harbouring the *siaD* gene as an EcoRI fragment derived from TopoKO0169	This study
pBBR[*siaD*^{R130A}]	pBBR[*siaD*] with the I-site altered from the functional RXXD- to the non-functional AXXD motif	This study
pBBR[*siaD*^{G140A}]	pBBR[*siaD*] with the A-site altered from the functional GGEEF to the non-functional GAEEF motif	This study
pUCP18-miniTn7t-[*rsmA*]	Delivery plasmid for insertion of the *rsmA* allele of *P. aeruginosa* under control of the pME6032 P_{BAD} promoter	Gift from Tina Jaeger from the Biozentrum,

	to the *att*Tn7 site	Basel
pTNS2	Plasmid for expression of the *tnsABCD* genes for mini-Tn7 transposition to *att*Tn7 site in *P. aeruginosa*	(Choi *et al.*, 2005)
pBBR[CC3396]	pBBR1-MCS5 harbouring the functional c-di-GMP specific phosphodiesterase CC3396 from *Caulobacter crescentus*	(Hickman *et al.*, 2005)
pUC18[*cupA1*]	pUC18 harbouring the template DNA for generating a *cupA1* sRNA fragment including 319 bp upstream of the start codon and a T7 transcription site	Gift from Tina Jaeger from the Biozentrum, Basel
pUC19[*rsmZ*]	pUC19 harbouring the template DNA for generating a *rsmZ* sRNA fragment including the complete *rsmZ* sequence and a T7 transcription site	This study
pUC19[*lolB*]	pUC19 harbouring the template DNA for generating a *lolB* sRNA fragment including 17 bp upstream of the start codon and a T7 transcription site	This study
pUC18[*cupA1*_AAA]	pUC18[*cupA1*] allele with a GGA to AAA mutation at nucleotides 309-311	This study
pME[*cupA*::*lacZ*]	pMP220 harbouring the *cupA1*::*lacZ* reporter construct (pMPFCAL), Tet^{r}	(Vallet *et al.*, 2004)
pCdrA::gfp^{C}	c-di-GMP reporter plasmid based on pUCP22 with the *cdrA* promoter driving *gfp*(Mut3) expression Ap^{r} Gm^{r}	Rybtke *et al.*, 2012
**Phage**		
E79tv2	Generalised transducing phage	(Morgan, 1979)

Supplementary Table S2. Primers used in the study.

**Plasmid construction**	
CupA F	GATCGGATCCGCG*TAATACGACTCACTATAGGG*AAGTCCAAACTGGCGAAGACAAGC
CupA R	GATCGAATTCTGCCGCCATCGCGGTTCCC
RsmZ F	GATCGGATCCGCG*TAATACGACTCACTATAGGG*CGTACAGGGAACACGCAACCC
RsmZ R	GATCGAATTCAAAAAAAAGGGGCGGGGTATTACCC
lolB F	GATCGGATCCGCG*TAATACGACTCACTATAGGG*GCAGCTCAGCGAGCTGGATG
lolB R	GATCGAATTCAGTTGTTCTTCCAGCAGTGCTTCCG
RsmA Nde F	ATTCATATGCTGATTCTGACTCGTCGG
RsmA His stop HindIII	TATAAGCTTTCAGTGATGGTGATG<br>GTGATGGTTTGGCTCTTGATC
**Quikchange**	
SiaD_A_F	GCCGCTGGGGCG**C**CGAGGAATTCCTC
SiaD_A_R	GAGGAATTCCTCG**G**CGCCCCAGCGGC
SiaD_I_F	GAGTCCGAGCTA**G**GCGGATACGACC
SiaD_I_R	GGTCGTATTCGC**C**TAGCTCGGACTC
CupA_AAA_F	GACGATTATTGCCTCACACAA**AA**AACATAGGCATGACCAGAAC
CupA_AAA_R	GTTCTGGTCATGCCTATGTT**TT**TTGTGTGAGGCAATAATCGTC
**qRT-PCR**	
cupA1 qPCR F	CGGCAAACACTATCACATTCAG

cupA1 qPCR R	CGGTACGCTGTCGAGGATC
siaD qPCR F	GCTGGCAATGCTCGATGTGGACTT
siaD qPCR R	CAGCGGCCGCAGAGGTCGTAT

Restriction endonuclease sites (underlined), T7 sequence for *in vitro* transcription (italic) and nucleotides muted (bold) are indicated.

Supplementary Table S3. Calculated molecular weight (MW) of all RNA fragments used in this study according to the Oligonucleotide Properties Calculator.

RNA	DNA length [nucleotides]	RNA length [nucleotides]	MW RNA [Dalton]
cupA1	440	397	108818.2
cupA1[AAA]	440	397	108786.1
rsmZ	162	119	38872.2
lolB	286	243	78751.2

References

Baba, T., Ara, T., Hasegawa, M., Takai, Y., Okumura, Y., Baba, M., *et al.* (2006). Construction of *Escherichia coli* K-12 in-frame, single-gene knockout mutants: the Keio collection. *Mol Syst Biol* 2**,** 2006 0008.

Bethesda, R.L. (1986). "BRL pUC host: *E. coli* DH5α^{TM} competent cells". Bethesda Research Lab.

Choi, K.H., Gaynor, J.B., White, K.G., Lopez, C., Bosio, C.M., Karkhoff-Schweizer, R.R., and Schweizer, H.P. (2005). A Tn7-based broad-range bacterial cloning and expression system. *Nat Methods* 2**,** 443-448.

Heeb, S., Blumer, C., and Haas, D. (2002). Regulatory RNA as mediator in GacA/RsmA-dependent global control of exoproduct formation in *Pseudomonas fluorescens* CHA0. *J Bacteriol* 184**,** 1046-1056.

Hickman, J.W., Tifrea, D.F., and Harwood, C.S. (2005). A chemosensory system that regulates biofilm formation through modulation of cyclic diguanylate levels. *Proc Natl Acad Sci U S A* 102**,** 14422-14427.

Kirisits, M.J., Prost, L., Starkey, M., and Parsek, M.R. (2005). Characterization of colony morphology variants isolated from *Pseudomonas aeruginosa* biofilms. *Appl Environ Microbiol* 71**,** 4809-4821.

Klebensberger, J., Birkenmaier, A., Geffers, R., Kjelleberg, S., and Philipp, B. (2009). SiaA and SiaD are essential for inducing autoaggregation as a specific response to detergent stress in *Pseudomonas aeruginosa*. *Environ Microbiol* 11**,** 3073-3086.

Kovach, M.E., Elzer, P.H., Hill, D.S., Robertson, G.T., Farris, M.A., Roop, R.M., 2nd, and Peterson, K.M. (1995). Four new derivatives of the broad-host-range cloning vector pBBR1MCS, carrying different antibiotic-resistance cassettes. *Gene* 166**,** 175-176.

Morgan, A.F. (1979). Transduction of *Pseudomonas aeruginosa* with a mutant of bacteriophage E79. *J Bacteriol* 139**,** 137-140.

Newman, J.R., and Fuqua, C. (1999). Broad-host-range expression vectors that carry the L-arabinose-inducible *Escherichia coli araBAD* promoter and the *araC* regulator. *Gene* 227**,** 197-203.

Rybtke, M.T., Borlee, B.R., Murakami, K., Irie, Y., Hentzer, M., Nielsen, T.E., *et al.* (2012). Fluorescence-based reporter for gauging cyclic di-GMP levels in *Pseudomonas aeruginosa*. *Appl Environ Microbiol* 78**,** 5060-5069. doi: 10.1128/AEM.00414-12.

Scheller, P.N., Fademrecht, S., Hofelzer, S., Pleiss, J., Leipold, F., Turner, N.J., *et al.* (2014). Enzyme toolbox: novel enantiocomplementary imine reductases. *Chembiochem* 15**,** 2201-2204.

Starkey, M., Hickman, J.H., Ma, L., Zhang, N., De Long, S., Hinz, A., *et al.* (2009). *Pseudomonas aeruginosa* rugose small-colony variants have adaptations that likely promote persistence in the cystic fibrosis lung. *J Bacteriol* 191**,** 3492-3503.

Vallet, I., Diggle, S.P., Stacey, R.E., Camara, M., Ventre, I., Lory, S., *et al.* (2004). Biofilm formation in *Pseudomonas aeruginosa*: fimbrial *cup* gene clusters are controlled by the transcriptional regulator MvaT. *J Bacteriol* 186**,** 2880-2890.

Valverde, C., Heeb, S., Keel, C., and Haas, D. (2003). RsmY, a small regulatory RNA, is required in concert with RsmZ for GacA-dependent expression of biocontrol traits in *Pseudomonas fluorescens* CHA0. *Mol Microbiol* 50**,** 1361-1379.
